# PReS13-SPK-1028: Activity and damage - we have to measure them

**DOI:** 10.1186/1546-0096-11-S2-I22

**Published:** 2013-12-05

**Authors:** H Brunner

**Affiliations:** 1 Pediatrics, Cincinnati Children's Hospital Medical Center, Cincinnati, USA

## Background

Childhood-onset SLE is a complex multiorgan disease. In order to judge the need of medical intervnetions and the patient benefits from them, measurement disease activity and damage are key. Furthermore, important improvement and deterioration of disease needs to be ascertained. Such measurement are the basis for clinical trial aimed at identifying improved medications and are needed to judge the benefits of medical interventions in general.

## Methods

An overview will be provided about current surrogate and biological markers of global and disease specific disease activity and damage. Focus will be placed on the relevance for children with SLE and current research activities, particularly NPSLE and lupus nephritis.

## Results & deliverables

Upon completion of the presentation the audience will h ave a firm understanding about the suitability of individual measures of disease activity and their differences. Measures include the various versions of the SLEDAI and BILAG, as well as SLE flare tools. The SLICC Damage Index and its pediatric version will be discussed in addition to previously validated measures of clinically relevant improvment and disease flare. Additionally, biomarkers of lupus nephritis as they are suited to help diagnose kidney disease and anticipate changes in the activity and chronicity of kidney lesions will be available. Some novel imaging biomarkers of neuropsychiatric damage will be reviewed.

## Disclosure of interest

None declared.

